# Does source population size affect performance in new environments?

**DOI:** 10.1111/eva.12181

**Published:** 2014-07-09

**Authors:** Matthew C Yates, Dylan J Fraser

**Affiliations:** Concordia UniversityMontreal, QC, Canada

**Keywords:** adaptation, conservation biology, meta-analysis, natural selection and contemporary evolution, population dynamics, population size, reciprocal transplant, translocation.

## Abstract

Small populations are predicted to perform poorly relative to large populations when experiencing environmental change. To explore this prediction in nature, data from reciprocal transplant, common garden, and translocation studies were compared meta-analytically. We contrasted changes in performance resulting from transplantation to new environments among individuals originating from different sized source populations from plants and salmonids. We then evaluated the effect of source population size on performance in natural common garden environments and the relationship between population size and habitat quality. In ‘home-away’ contrasts, large populations exhibited reduced performance in new environments. In common gardens, the effect of source population size on performance was inconsistent across life-history stages (LHS) and environments. When transplanted to the same set of new environments, small populations either performed equally well or better than large populations, depending on life stage. Conversely, large populations outperformed small populations within native environments, but only at later life stages. Population size was not associated with habitat quality. Several factors might explain the negative association between source population size and performance in new environments: (i) stronger local adaptation in large populations and antagonistic pleiotropy, (ii) the maintenance of genetic variation in small populations, and (iii) potential environmental differences between large and small populations.

## Introduction

The management of small populations remains a major focus of conservation biology. Habitat fragmentation due to ongoing anthropogenic activities has resulted in the depletion of many species, such that many now exist only as small, isolated populations. Population size is thought to be associated with risk factors that impact the capacity of populations to persist in a changing environment (Willi et al. 2006; Frankham et al. 2014). In addition to an increased risk of extinction due to demographic and environmental stochasticity (Lande 1988; Frankham 2005), reduced genetic diversity and the exposure of accumulated deleterious alleles at small population size could result in genetic Allee effects that diminish the capacity of small populations to persist under environmental change (Lynch and Lande 1993; Leimu et al. 2006; Willi et al. 2006; Bowman et al. 2008; Bijlsma and Loeschcke 2012).

Previous studies of natural populations have found positive relationships between population size and fitness components (e.g., Reed 2005; Leimu et al. 2006). However, these studies were largely based on observational measurements of populations in their local environments or artificial common garden experiments (Oakley 2013). For several reasons, the extent to which the observed increased fitness in large populations might translate into enhanced persistence under changing or novel environmental conditions remains unclear. First, the strength of local adaptation is positively associated with population size (Leimu and Fischer 2008), so observational studies that measure fitness solely within native environments could be confounded by this effect. Second, some forms of local adaptation involve antagonistic pleiotropy, wherein alleles that are favored in a population's local environment reduce fitness in other environments (Kawecki and Ebert 2004). Under such antagonistic pleiotropy, stronger local adaptation in large populations might actually reduce performance under changing environmental conditions. Third, small populations may inhabit marginal environments (Hoffmann and Blows 1994; Kawecki 2008). Observational studies comparing fitness components between large and small populations may be confounded by a systematic bias in habitat quality (Oakley 2013). Fourth, a previous history of adaptation to marginal stressful environments may enhance performance in novel environmental conditions (Reed et al. 2003; Gonzalez and Bell 2012). Finally, the relatively benign conditions in artificial common garden environments may not be representative of typical stresses found in nature.

Small populations might perform poorly in novel environmental conditions due to low levels of genetic variation and an increased number of fixed deleterious mutations as a result of inbreeding (Willi et al. 2006; Oakley 2013). However, while population size is positively correlated with neutral genetic diversity (Reed and Frankham 2003), neutral genetic diversity is weakly correlated with quantitative genetic variation (Reed and Frankham 2001; Ouborg et al. 2006). Existing empirical studies in nature rarely report strong correlations between population size and quantitative genetic variation or heritability in wild populations (Willi et al. 2006). Furthermore, under some forms of selection, population size may not have a significant effect on genetic variation except at extremely small sizes (Willi et al. 2006). Finally, in plants, there is evidence that the magnitude of detrimental inbreeding effects is positively associated with population size, indicating that some small populations may evolve some resistance to inbreeding depression (Angeloni et al. 2011).

In the absence of information tracking how populations adapt to change within their native environment over successive generations, replicated translocations to novel natural environments of subsamples of individuals from varying sized source populations represent an opportunity to discern the possible effect that source population size has on performance under environmental change. Those few studies that have attempted such translocations have yielded inconsistent results. Small populations either (i) outperformed large populations (Hooftman et al. 2003), (ii) exhibited no loss of fitness or were outperformed by larger populations only in more benign environmental conditions (Oakley 2013), or (iii) exhibited reduced performance in increasingly dissimilar environments relative to their native environment (Bowman et al. 2008). Collectively, the effect of source population size on performance under natural environmental conditions merits further investigation before general inferences can be made.

Our meta-analysis is a first attempt on multiple taxa to directly test, in nature, the prediction that larger source population size improves the performance of individuals transplanted to novel environments, while simultaneously accounting for possible confounding relationships between population size and local adaptation or habitat quality.

We specifically conducted three separate analyses. The first evaluated how the performance of individuals from source populations of known size changed in novel environments. We performed a ‘home-away’ contrast analysis that compared the performance of individuals within a populations’ native environment to the performance of individuals translocated to a novel environment. Relevant data were obtained principally from reciprocal transplant studies and translocation experiments.

The second related ‘common garden’ analysis was conducted on data from common garden experiments in which randomly sampled individuals from source populations of known size were transplanted to the same set of natural novel environments (this included reciprocal transplants). By doing so, this analysis controlled for any potential confounding relationships between population size and the strength of local adaptation or habitat quality on performance not accounted for in the ‘home-away’ contrast above.

Finally, the third ‘habitat quality’ analysis used data exclusively from reciprocal transplants to determine whether large populations tended to inhabit better-quality environments. By comparing the survival of individuals from the same set of populations within the same set of environments, this analysis could assess whether survival across these environments was associated with the size of the populations naturally inhabiting them, while controlling for the effect of local adaptation and source population size on survival.

## Materials and methods

### Quantitative review of primary literature

We conducted keyword searches on the academic search engine ISI Web of Science™. A complete keyword search of ‘local* adaptation*’ + ‘reciprocal* Transplant*’ was performed, as well as for the phrases ‘phenotyp*’ + ‘plastic*’ + ‘Transplant*’. References within studies were then used to obtain studies missed by keyword searches, with emphasis on other reciprocal transplants and meta-analyses.

Survival was chosen as a relative fitness component for our three analyses due to its relatively unambiguous relationship with fitness and ease of standardization across studies. Only populations for which survival data and measurements of adult census population size could be found were included in the analysis. While suitable transplant experiments were quite common in plants, few of these experiments have been conducted on vertebrates outside of salmonid fishes; all suitable vertebrate studies found were conducted on salmonids.

Many transplant studies reported survival in both native and novel (‘away’) environments, but lacked data on source population size, whereas others reported population size but lacked survival data. For many studies, source population size data were found using other resources (journal publications, government databases, etc.), particularly for well-studied salmonids. If relevant fitness or population size data were unobtainable in the original paper or through secondary sources, primary and secondary authors were directly contacted to obtain the information. When survival and/or population size information was contained in figures, the program ImageJ (Abramoff et al. 2004) was used to extract relevant data. Finally, if multiple years of population size data existed for a population, the harmonic mean was used.

### Testing performance in new environments using home-away contrasts

To test how source population size affects the performance of a population in a novel environment relative to its native environment, the survival of transplanted individuals from populations of known census size was compared in ‘home’ and ‘away’ environments. Although this only compares the performance of single populations across multiple environments, it is meant to assess the capacity of individual populations to respond to new environments regardless of the performance of other populations in those environments.

The effect2 size of the relative proportions of surviving individuals in the home-away contrast was calculated for each population using the log odds ratio (Lipsey and Wilson 2001), represented by the following equation:


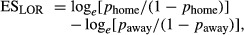


where ES_LOR_ is the log odds ratio effect size, *p*_home_ was the proportion of individuals surviving in their home environment, and *p*_away_ was the proportion of individuals surviving in the transplant environment. A positive effect size value indicates better performance in the home relative to the novel environment, a negative effect size value the converse. For any comparisons with zero survival in either the home or transplant environment, a value of 0.5 was added to these cells; conversely, 0.5 was subtracted in any environment with 100% survival (Lipsey and Wilson 2001). This particular manipulation of the data tends to create a downward bias and at worst will provide conservative estimates of the effect size statistic (Lipsey and Wilson 2001). Comparisons involving zero survival in both environments were excluded.

A formal, mixed-effects meta-analysis was conducted using a generalized linear mixed-effects (GLMM) model with ES_LOR_ as the dependent variable in the analysis and weighted based on inverse variance weights. As genetic variation is nonlinearly related to population size (Willi et al. 2006) and the detrimental effects of inbreeding are severe only at extremely small population sizes (Jamieson and Allendorf 2012), the log_10_ of the size of the source population was included as a fixed continuous covariate. To test how performance in novel environments could be affected by life history or evolutionary characteristics, two other categorical fixed effects were included: (i) the transplanted population's taxa (salmonid or plant) and (ii) the life-history stage of the transplanted organism (embryonic/postembryonic stage versus a later life-history stage; e.g., germination versus seedling transplants for plants or fry versus fingerling/smolt releases for salmonids), as this can affect subsequent performance in plants and salmonids (Raabova et al. 2007; Fraser 2008). All interactions between fixed effects were tested.

Species, population, and transplant site were included as random effects in all models to control for issues of nonindependence (pseudoreplication) arising from multiple comparisons. Many species and populations included in our study were examined at multiple LHS, so random effects were conditioned on life-history stage. Although study is typically included in meta-analysis as a random effect, it was omitted here because of its almost complete correlation with species (few studies examined the same species) and because most studies examining the same species were conducted by the same researchers.

To assess the effect of source population size on performance in novel environments, a formal meta-analysis was conducted using the MCMCglmm package (Hadfield 2010) in R 3.0.2 (R Core Team 2013). The analysis was initiated using a full model that included all fixed and random effects. Fixed effect parameters were removed in a stepwise fashion, using Deviance Information Criterion (DIC) to evaluate model fit (Spiegelhalter et al. 2002). All random effects were retained in each model, regardless of significance. The default (weakly informative) priors were used for each run, which had a burn-in phase of 100 000, a thinning interval of 20, and 500 000 iterations. Alterations to priors (e.g., *V* = 1, *ν* = 0.002) did not significantly affect model conclusions.

### Testing the effect of source population size on survival in natural common garden environments

If the previous statistic (ES_LOR_) is solely used, it is possible that one population might exhibit greater performance in all environments relative to another transplanted population but exhibit a reduced effect size (i.e., worse survival in its home environment relative to the transplant environments). That is, comparing a population's performance in transplant environments relative to its performance in its home environment does not control for a population's overall performance relative to others. We therefore also collated and analyzed the survival of individuals from multiple source populations of known size that were transplanted to novel common garden natural environments, including reciprocal transplants.

Survival was assessed in relation to possible explanatory variables as a binomial variable using a GLMM with a logit-link function. The analysis was conducted using the function *glmer* in the statistical package *lme4* (Bates et al. 2012) in R 3.0.2. The log_10_ of population size was included as a continuous fixed covariate. Life-history stage was included as a categorical fixed effect, as was a ‘local versus foreign’ contrast to account for differences in survival associated with local adaptation to home environments. All possible interactions were included as fixed effects. Taxon was not included in this analysis due to a lack of common garden experiments among salmonids. Species, population, and transplant environment were included as random effects conditioned on life-history stage to account for any nonindependence in the data. Observation-level random effects were fitted to the model to account for issues of overdispersion (Browne et al. 2005).

Model fit was evaluated using Akaike's Information Criterion (AIC) (Akaike 1974), corrected for small sample size bias (AIC_c_) (Hurvich and Tsai 1989). Model selection was first conducted by stepwise reducing random effect terms, although intercept effects were retained regardless of fit. Fixed effects terms were then stepwise removed, eliminating interaction effects first. If an interaction was significant, all relevant lower-order terms were retained. Once a best-fit model was obtained, Wald *χ*² tests were used to evaluate the significance of fixed effect terms and Wald *Z*-tests were used to evaluate the significance of pairwise contrasts between term levels.

### Testing if large populations tend to inhabit better-quality environments

To assess the potential relationship between habitat quality and population size that may have confounded previous estimates of population size and fitness (Oakley 2013), a third analysis was conducted on the subset of populations involved in reciprocal transplant experiments. In reciprocal transplants, every population is transplanted to every other population's native environment. The consistent use of multiple populations across environments provided an unbiased estimate of overall survival within each environment that could control for potential confounding effects of source population size and local adaptation on performance.

To test whether large populations tended to inhabit higher-quality environments, we assessed the correlation between overall survival in environments within reciprocal transplants and the size of the populations naturally inhabiting those environments. Survival was assessed as a binomial variable using a GLMM with a logit-link function. Analysis was conducted with the function glmer in the statistical package *lme4* (Bates et al. 2012) in R 3.0.2. Both the log_10_ of the size of the source population of the transplanted populations and the log_10_ of population size of the transplant site population were included as fixed continuous covariates. Life-history stage was also included as a categorical fixed effect, as was a ‘local-foreign’ contrast to account for differences in survival due to local adaptations. All possible interactions, with the exception of interactions involving the size of the population inhabiting the environment and source population size or a local-foreign contrast, were included in the initial model. Species, population, and transplant environment were included as random effects conditioned on life-history stage to account for nonindependence in the data. Observation-level random effects were fitted to the model to account for issues of overdispersion (Browne et al. 2005). Model selection proceeded as described for the natural common garden analysis.

## Results

### Summary of meta-analysis data

Our meta-analysis contained 874 estimates of survival from 111 populations ranging in population size from 9 to 100 000 individuals (median = 400), of which 102 populations were from plants and 9 from salmonids (13 total species; Table 1); no suitable studies with population size data were found for other taxa. The first ‘home-away’ contrast dataset was comprised of 88 populations of plants and salmonids (Table 1). The second ‘common garden’ dataset included data on 100 plant populations (including reciprocal transplants; mean number of populations per experiment = 10; Table 1). The third ‘habitat quality’ dataset was constructed with 53 plant populations from reciprocal transplant studies (Table 1).

**Table 1:** Summary of survival data for populations of known size transplanted to novel environments

							Home versus away*			
								
Species	Taxa	Populations	Transplant type	Subanalysis used	Life-history stage	Total transplants	>	=	<	References
*Arabidopsis thaliana*	Plant	2	Reciprocal	All	Late	8	2	–	2	Callahan and Pigliucci (2002)
*Hypochoeris radicata*	Plant	10	Reciprocal	All	Late	34	6	15	3	Becker et al. (2008)
*Inula hirta*	Plant	6	Reciprocal	All	Both	72	21	29	10	Raabova et al. (2011)
*Armeria elongate*	Plant	24	Common garden translocation	Home versus away, common garden	Early	175	34	135	15	Seifert and Fischer (2010)
*Arabidopsis lyrata*	Plant	8	Common garden translocation	Common garden	Late	32	NA	NA	NA	Vergeer and Kunin (2013)
*Carlina vulgaris*	Plant	23	Reciprocal	All	Both	108	17	41	22	Jakobsson and Dinnetz (2005) and Becker et al. (2006)
*Aster amellus*	Plant	12	Reciprocal	All	Both	351	48	184	29	Raabova et al. (2007, 2008)
*Purshia subintegra*	Plant	1	Translocation	Home versus away	Late	4	–	–	3	Maschinski et al. (2004)
*Scorzonera humilis*	Plant	1	Reciprocal	Home versus away	Early	12	1	5	5	Reckinger et al. (2010)
*Hypericum cumulicola*	Plant	15	Common garden translocation	Common garden	Late	30	NA	NA	NA	Oakley (2013)
*Salmo salar*	Salmonid	3	Reciprocal, translocation	Home versus away	Both	23	7	10	0	Ritter (1975)† and Houde et al. (2011)†
*Oncorhynchus kisutch*	Salmonid	4	Translocation	Home versus away	Early	10	5	–	1	Bagatell et al. (1980)§; Bagatell et al. (1981)§ and Fuss and Rasch (1981)§
*Oncorhynchus tshawytscha*	Salmonid	2	Translocation	Home versus away	Both	15	2	1	5	Federenko and Shepherd (1986) and Unwin et al. (2003)

* ‘>’ indicates statistically better performance in the home environment, ‘=’ indicates no statistical difference between performance in the ‘home’ and ‘away’ environments, and ‘<’ indicates when a population performed statistically better in the ‘away’ environment. Measurements where survival was zero in both home and away environment not included. NA refers to common garden experiments which lack a comparison in home environments and were thus not used for the ‘home versus away’ meta-analysis.

† Population size data obtained from DFO (2012) and Douglas et al. (2013).

‡ Population size data obtained from Gibson and Amiro (2003).

§ Population size data obtained from SalmonScape, published by the Washington Department of Fish and Wildlife (2012).

### Effect of population size, life-history stage, and taxa on relative performance using home-away contrasts

The best fit model included only source population size as a fixed effect. The inclusion of other parameters did not improve model fit (Table 2) or change the significance of fixed effects terms. Although a simpler intercept-only model had a close DIC value (ΔDIC = 1.08), population size was retained as a fixed effect due to its statistical significance and improved fit.

**Table 2:** Best fit MCMCglmm models (evaluated using Deviance Information Criterion, DIC) predicting performance in novel environments relative to a population's native environment. LHS refers to life-history stage, *N* refers to log_10_ source population size, and Taxa refers to whether the transplant was a salmonid or plant

Model	DIC	ΔDIC
*N*	1476.218	0.0
*N* + LHS	1476.803	0.585
*N* + LHS + Taxa	1477.153	0.935
Intercept-only	1477.302	1.084
*N* + Taxa	1477.420	1.202

**Figure 1:**
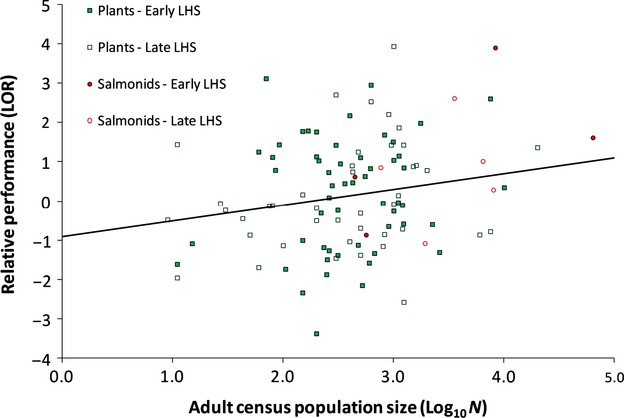
The effect of log_10_ census population size on the average survival of a population in novel (‘away’) environments relative to its native environment. Solid squares, plants, early life-history stages (LHS); Open squares, plants, later LHS; Solid circles, salmonids, early LHS; Open circles, salmonids, later LHS.

Source population size had a negative effect on relative performance in novel environments. As source population size increased, transplanted populations exhibited reduced performance in novel environments relative to their native environment (*P*_mcmc_ = 0.020, Fig. 1).

### The effect of population size on overall performance in natural common garden environments

The best fit model describing overall performance in natural common garden environments included all random effects, fixed effects, and two-way interactions (AICc = 4186.69, Table 3). There was some support for the removal of an interaction between the effect of source population size and local–foreign contrast (ΔAICc = 1.41) and the effect of source population size and life-history stage (ΔAICc = 1.49). However, both subsequent models had similar weights, the further removal of terms did not improve model fit, and both interaction terms exhibited statistical significance or marginal significance, so both interactions were retained. As in previous studies of local adaptation (Hereford 2009, Fraser et al. 2011), populations exhibited significantly better performance in their native environment relative to novel environments (*χ²* = 10.679, *P* = 0.001, Table 4). However, this depended on the life-history stage of the transplanted organisms (*χ²* = 5.756, *P* = 0.016). Evidence was also found that the effect of source population size depended upon the life-history stage of the transplants (*χ²* = 3.993, *P* = 0.046, Table 4) and whether they were transplanted to a novel environment or their native environment (*χ²* = 3.580, *P* = 0.058).

**Table 3:** The six best fit GLMM models (evaluated using Akaike's Information Criterion, AIC_c_) predicting overall performance in common garden experiments conducted in natural environments. LHS refers to life-history stage, *N* refers to log_10_ source population size, and Local refers to whether a population was transplanted to its native environment or a foreign environment

Model	AIC	AIC_c_	ΔAIC	wAIC
*N* + LHS + Local + LHS:Local + *N*:LHS + *N*:Local	4185.9	4186.69	0	0.390
*N* + LHS + Local + LHS:Local + *N*:LHS	4187.4	4188.10	1.41	0.193
*N* + LHS + Local + LHS:Local + *N*:Local	4187.3	4188.19	1.49	0.185
Full model	4187.7	4188.40	1.71	0.166
*N* + LHS + Local + LHS:Local	4189.5	4190.12	3.42	0.067

**Table 4:** Analysis summaries of overall performance in common garden experiments performed in natural environments and the relationship between population size and habitat quality. Survival, expressed as a binomial variable, was used as the response. Only results for the best fit models are presented. LHS refers to life-history stage, *N* refers to log_10_ source population size, NTrans refers to the log_10_ size of the population naturally inhabiting a transplant site, and Local refers to whether a population was transplanted to its native environment or a foreign environment

	Overall performance		Habitat quality versus *N*	
		
Predictor	*χ²*	*P*-value	*χ²*	*P*-value
*N*	0.040	0.841	0.200	0.655
LHS	20.355	<0.001	8.157	0.004
Local	10.679	0.001	10.584	0.001
*N*:Local	3.580	0.058	4.492	0.034
*N*: LHS	3.993	0.046	4.740	0.029
LHS:Local	5.756	0.016	5.125	0.024

At early LHS, transplanted organisms exhibited improved performance in native habitats relative to novel environments. We found some evidence that this was a result of a performance cost associated with source population size exhibited only in novel environments (*Z* = 1.915, *P* = 0.055, Table 5), although this trend was only marginally different relative to the effect of source population size on performance in native environments (*Z* = 1.897, *P* = 0.058). When transplanted to their native habitat at early LHS, all populations, regardless of source size, performed equally well (*Z* = 0.158, *P* = 0.875, Table 5).

**Table 5:** Effect of log_10_ source population size (*β*) on performance in novel and native environments at different life-history stages (LHS). Units are in log odds

LHS and environment	Intercept	*β*	SE (*β*)	Z	*P*-value
Early LHS, novel	−2.999	−0.2727	0.1424	−1.915	0.055
Early LHS, native	−3.238	0.0310	0.1964	0.158	0.875
Later LHS, novel	0.383	0.0600	0.0889	0.674	0.500
Later LHS, native	−0.313	0.3578	0.1573	2.274	0.023

The effect of source population size differed for organisms transplanted at later LHS relative to those transplanted at earlier stages (Z = 1.998, *P* = 0.046). When organisms were transplanted at later LHS to their native environments, source population size had a positive effect on performance that was significantly different from zero (*Z* = 2.274, *P* = 0.023, Table 5). Despite this association, no evidence was found that organisms at later LHS exhibited local adaptation due to a significantly lower intercept value in native environments relative to earlier LHS (*Z* = 2.399, *P* = 0.016). Although a trend was observed that small populations exhibited maladaptation and large populations exhibited local adaptation at later LHS, neither large nor small populations exhibited significantly different overall performance in native relative to novel environments. In novel environments, the effect of source population size on performance at later LHS was small and not statistically different from zero (*Z* = 0.674, *P* = 0.500, Table 5), but was only marginally different relative to its effect on performance in native environments (*Z* = 1.897, *P* = 0.058).

The life-history stage of the transplanted organisms also had a significant overall influence on performance; plants transplanted at later LHS exhibited improved performance (*χ²* = 20.355, *P* < 0.001, Table 4).

### Do large populations tend to inhabit better-quality habitat?

The best fit model evaluating habitat quality contained all random effects, all fixed effects except for transplant site population size, and all subsequent two-way interactions. (AICc = 2960.24, Table 6). There was some evidence for the removal of the interaction between source population size and the local-foreign contrast (AICc of 2960.48 versus 2961.84, Table 6). However, for similar reasons as described in the common garden analysis, the more complex model was retained. There was also some evidence to support the inclusion of the transplant site population size term (ΔAIC = 0.23). However, this term was not significant and was subsequently removed.

**Table 6:** The six best fit GLMM models (evaluated using Akaike's Information Criterion, AIC_c_) predicting the relationship between habitat quality and population size. Analysis was conducted using generalized linear mixed-effects models in *lme4*. LHS refers to life-history stage, *N* refers to source population size, NTrans refers to the log_10_ size of the population naturally inhabiting a transplant site, and Local refers to whether a population was transplanted to its native environment or a foreign environment

Model	AIC	AIC_c_	ΔAIC	wAIC
Local + LHS + *N* + *N*: LHS + *N*:Local + Local: LHS	2959.1	2960.24	0.0	0.339
NTrans + Local + LHS + *N* + *N*: LHS + *N*:Local + Local: LHS	2959.2	2960.48	0.23	0.301
NTrans + Local + LHS + *N* + *N*: LHS + Local: LHS	2960.7	2961.84	1.60	0.152
Full model	2961.0	2962.43	2.18	0.114
NTrans + Local + LHS + *N* + *N*:Local + Local: LHS	2961.7	2962.84	2.60	0.093

When only reciprocal transplants were examined, the relationships between performance and source population size, life-history stage, and local adaptation remained consistent with the previous analysis or increased in strength. Populations exhibited local adaptation (*χ²* = 10.584, *P* = 0.001), but this was dependent upon the life-history stage of the transplant (*χ²* = 5.125, *P* = 0.024). The effect of source population size also depended upon the life-history stage of the transplants (*χ²* = 4.740, *P* = 0.029) and whether they were transplanted to a novel environment or their native environment (*χ²* = 4.492, *P* = 0.034).

In reciprocal transplant experiments, only early life-history stage transplants exhibited local adaptation. Similar to the previous analysis, this was a due to a negative effect of source population size on performance in novel environments at early LHS (*Z* = 2.493, *P* = 0.013). The effect of source population size on transplanted organisms differed between native and novel environments (*Z* = 2.115, *P* = 0.035), with source population size having no effect on performance at early LHS within native environments (*Z* = 0.475, *P* = 0.635). Source population size had a positive effect on performance in native environments at later LHS in reciprocal transplants (*Z* = 2.253, *P* = 0.0243). However, organisms transplanted at later LHS exhibited no effect of population size on performance in novel environments (*Z* = 0.054, *P* = 0.957). No evidence was also found that the performance of organisms transplanted at later LHS differed between native and novel environments.

## Discussion

### Effect of source population size on performance in novel environments

In home-away contrasts, individuals from large source populations experienced greater reductions in performance in novel environments than those from smaller populations. As ES_lor_ was based on the relative performance of a population in a novel environment compared to within its native environment, we cannot discern whether the decreased performance of large populations in novel environments is a result of stronger local adaptation in their native environments (e.g., Leimu and Fischer 2008), poor overall performance in novel environments, or a combination of the two. At the very least, our results indicate that large populations experienced greater declines in fitness relative to smaller populations when exposed to novel environmental change.

By examining the performance of multiple populations in natural common gardens and reciprocal transplants, however, we were able to further clarify some aspects of the relationship between population size and performance. Common garden experiments allowed us to control for confounding effects if fitness is only examined observationally in each population's native environment (Oakley 2013) or through home-away comparisons. Similar to our home versus away analysis, we found that large populations tended to exhibit improved performance in their native environments relative to novel environments. However, the effect of source population size on overall performance was inconsistent across LHS and transplant environments: in novel environments, large source population size was associated with a marginal performance cost at early LHS but had no effect at later LHS. Conversely, in native environments, large source population size had no effect on performance at early LHS but had a significant positive effect on performance at later LHS, although we found no overall evidence of local adaptation at this life-history stage. The finding that large source population size had either no effect or a negative effect on performance in novel environments runs counter to some theoretic predictions that small populations are expected to exhibit reduced performance in stressful conditions due to potential genetic Allee effects (Reed and Frankham 2003; Leimu et al. 2006; Bijlsma and Loeschcke 2012). Inbreeding, in particular, is thought to be exacerbated in stressful conditions (Fox and Reed 2010), but we found evidence that small populations either performed as well as or slightly better than large populations when transplanted to the same set of natural novel environments.

### Effect of taxa on performance in novel environments

Although comparative taxonomic data were limited to our home-away contrasts, we found no evidence that relative performance in novel environments differed between plants and salmonids. Data required for such taxonomic comparisons are still rare in the literature; despite being a well-studied species group, we found population size information for only nine salmonid transplants. Nevertheless, the extent of local adaptation in salmonids has been estimated to be similar to plants (Fraser et al. 2011), so a lack of differentiation between these two groups was not unexpected.

### Is population size positively associated with habitat quality?

Previous studies examining the relationship between population size and fitness have largely relied on observational field studies (e.g., Leimu et al. 2006), which cannot account for potential differences in habitat quality and local adaptation. However, we found no evidence that overall survival differed in environments naturally harboring small or large populations. Our analysis was conducted on a subset of population data used in the common garden analysis (reciprocal transplants only). While the sample size for this analysis was the smallest of the three (only 53 populations), all other results were similar to those obtained from the analysis conducted on all common garden environments.

### Potential caveats

When relating population size to genetic variation, the effective population size (*N*_e_), not adult census population size, is the most appropriate measurement to use (Angeloni et al. 2011). Estimates of *N*_e_ were not available for any populations in our meta-analysis. Yet based on empirically estimated *N*_e_/*N* ratios in nature (Frankham 1995; Palstra and Fraser 2012), we can infer that many of the small populations included in our meta-analysis had *N*_e_ well under 50 (minimum population size in our study = 9), below which populations should experience significantly low levels of genetic variation and detrimental levels of inbreeding (Willi et al. 2006; Frankham et al. 2014). In other words, if *N*_e_ was positively correlated with a population's performance in new environments, survival reductions in small populations would still have been observed.

Our conclusions are also based on data from plants and salmonids; the extent to which they can be generalized to other taxa is unclear. Nevertheless, our meta-analysis included 874 estimates of survival from 111 populations across 13 species and also covered a large range of census population sizes (between 9 and 100 000). Furthermore, the large number of populations sampled relative to the number of species may help control for variation in the response to novel environments.

### Possible explanations for elevated performance of small populations

Why did we find evidence that small populations exhibited similar or slightly better performance relative to large populations when transplanted to novel natural environments, when previous analyses based on observational studies or artificial common gardens have found significant positive relationships between source population size and fitness (e.g., Reed 2005; Leimu et al. 2006)? We propose three hypotheses. These raise a number of points meriting further discussion and empirical consideration, and they relate to: (i) the potential effect of population size on the strength of local adaptation and subsequent pleiotropic trade-offs (ii) the maintenance of genetic variation in small populations; and (iii) other potential systemic differences in habitat between large and small populations.

#### Population size in relation to the strength of local adaptation

Previous research found that population size was positively associated with the strength of local adaptation (Leimu and Fischer 2008). We contend that results from our meta-analysis are consistent with this observation. In our natural common garden analysis, significant local adaptation was only exhibited at early life-history stages, at which local adaptation is thought to be strong in plants (Raabova et al. 2007 and references therein). We found marginal evidence that this resulted from a negative correlation between source population size and performance in novel common garden environments. Antagonistic pleiotropy can underlie local adaptations (Kawecki and Ebert 2004; Anderson et al. 2013), so if large populations exhibit stronger local adaptation, they may initially exhibit reduced performance in novel environmental conditions. However, a concomitant increase in the association between population size and performance within native environments should also have been observed if the negative relationship between source population size and performance in novel environments resulted from antagonistic pleiotropy. Instead, at early LHS, individuals from populations of all sizes exhibited similar performance within their native environments.

Due to the inherent design of the experiments used in the common garden analysis, our capacity to detect the effect of source population size on performance within native environments was limited relative to our capacity to detect trends in ‘novel’ environments. The quantity of information available on the performance of a population in novel environments will exceed that available on their performance in their native environment in reciprocal transplants involving more than two populations. Additionally, due to the inclusion of nonreciprocal common garden transplants in our dataset, survival data for transplanted populations in their native environments were only available for 53 of the 100 populations analyzed, and of those, only 29 populations had early life-history stage data available. Our capacity to detect benefits associated with local adaptation may have been reduced relative to our capacity to detect antagonistic pleiotropic costs, particularly if the magnitude of those benefits is lower than the fitness costs exhibited in novel environments.

Despite these limitations, our data potentially suggest that the costs and benefits of local adaptation could be experienced during different LHS. Although we found no overall evidence of significant local adaptation at large source population sizes (or maladaptation at small source population sizes) during later LHS, we did find a statistically significant association between source population size and performance in later LHS that was exhibited within native environments. This finding is consistent with previous results from observational studies that found positive associations between population size, fitness, and local adaptation in wild populations in their native habitats (Reed 2005; Leimu et al. 2006; Leimu and Fischer 2008) and could suggest an improved capacity among large populations to locally adapt to their native environments.

#### Genetic variation and isolation in small populations

Small populations exhibited similar or better performance relative to large populations in novel common garden environments, providing no evidence of genetic Allee effects resulting from reduced genetic diversity, increased inbreeding, and increased genetic load (Willi et al. 2006; Bowman et al. 2008). Although increased local adaptation in large populations and resulting antagonistic pleiotropy could account for some of this relationship, several processes might act to retain genetic variation in small natural populations, buffering them against the negative genetic effects of small population size. Purging may be more efficient in some smaller plant populations (Angeloni et al. 2011), resulting in a lower genetic load when faced with environmental change. Furthermore, gene flow may buffer some small populations against a loss of genetic diversity (Willi et al. 2006). The extent of migration in many of the study populations is relatively unknown. The potential for asymmetric gene flow between large and small populations could constrain local adaptation in small populations (Ellstrand 1992) yet simultaneously alleviate the detrimental effects of inbreeding (Frankham 2005).

#### Systemic differences in environments between large and small populations

If large and small populations inhabit environments that vary systemically, previous observational studies examining the relationship between population size and fitness may potentially be confounded. While we did not find any association between habitat quality and population size, habitat may vary systematically between large and small populations in other ways. Habitats inhabited by small populations may tend to be more variable, for example (Wood et al. 2014), potentially resulting in increased phenotypic plasticity in smaller populations that could confer tolerance to environmental change.

### Conclusions and future research directions

Our meta-analysis raises important questions about the nature of commonly observed fitness trade-offs in local adaptation studies (Hereford 2009) and how they might relate to population size. Specifically, what is the magnitude of the cost of such trade-offs? Is a fitness increase in a population's native environment associated with an equal reduction in fitness in novel environments, or is it associated with a disproportionate fitness decline in novel environments? How are the costs and benefits of fitness trade-offs distributed across LHS?

We found some evidence that source population size was associated with decreased performance in novel environments during life-history stages at which local adaptation is strong. However, because of limited data in the literature, we cannot presently conclude whether the performance of large populations in their native environments was compensated by increased local adaptation, although we postulate that it is likely based on related findings in previous studies (e.g., Reed 2005; Leimu et al. 2006; and Leimu and Fischer 2008).

We also found no evidence for potential genetic Allee effects associated with small population size in novel environments. Under some novel selection regimes, small populations appear to cope with short-term environmental change as well as – or better than – large populations. Whether this also translates into enhanced long-term persistence is unknown: the potential for increased genetic diversity in larger populations may allow them to better adapt to novel change over subsequent generations than small populations, despite an initially larger demographic impact. Many organisms can respond to environmental change through rapid adaptation, in which case large population size may play a significant and important role (i.e., Samani and Bell 2010). However, it is important to note that for species with long generation times, the capacity of individuals to tolerate environmental change may facilitate their persistence under novel environmental conditions.

Furthermore, the widespread distribution and/or generalist nature of most of the species in our study could affect the influence of population size on performance in new environments. Generalist species that are capable of tolerating a wide range of environments may be buffered against environmental change through phenotypic plasticity and/or could be capable of persisting at small population sizes due to nonevolutionary responses. Conversely, specialist species that occupy narrow niches and limited geographic ranges are already vulnerable to environmental disturbance and prone to extinction (Kotiaho et al. 2005). While the small number of species in our study precluded our ability to test for the effect of common versus rare distributions or generalist versus specialist strategies, these may affect the relative importance of population size on performance.

Future research into the effect of source population size on the strength of local adaptation and performance in novel, natural environments should endeavor to focus on the magnitude of trade-offs associated with local adaptation at multiple LHS. Additional research into the performance of subsequent generations in transplant environments could assess the long-term adaptive consequences of source population size and its effect on genetic variation, an issue of particular relevance for both the conservation of threatened species and invasive species biology (Theoharides and Dukes 2007; Frankham et al. 2014).

Reciprocal transplants represent the best research designs available to control for potential confounding effects that could influence estimates of the effect of source population size and may also allow researchers to disentangle the magnitude of trade-offs associated with local adaptation. We would encourage future reciprocal transplant experiments to include, when possible, population size estimates.

## Data archiving statement

Data for this study is available from the Dryad Digital Repository: http://dx.doi.org/10.5061/dryad.81k4v.
